# Obituary

**Published:** 1910-01-08

**Authors:** 


### OBITUARY.

The death is announced of the Hon. Arthur Hill Trevor de Montmorency, M.D., L.R.C.S.I., near Dublin, in his 64th year. Mr. de Montmorency became a Bachelor of Medicine in 1873 and took the degree of M.D. two years later. Dublin was his University, and when he gave up practice he turned with great success to horticulture.

Lieut.-Col. Campbell Mellis Douglas, V.C., M.D., R.A.M.C., is reported to have died at his home in Somerset at the age of 69. He became an L.R.C.S. of Edinburgh, and took his degree of M.D. at the same University in 1861.

